# No association between blood-based markers of immune system and migraine status: a population-based cohort study

**DOI:** 10.1186/s12883-023-03496-w

**Published:** 2023-12-18

**Authors:** Cevdet Acarsoy, Rikje Ruiter, Daniel Bos, M. Kamran Ikram

**Affiliations:** 1 Department of Epidemiology, Erasmus MC, University Medical Center Rotterdam, Rotterdam, the Netherlands; 2 Department of Internal Medicine, Maasstad Hospital, Rotterdam, the Netherlands; 3 Department of Radiology and Nuclear Medicine, Erasmus MC, University Medical Center Rotterdam, Rotterdam, the Netherlands; 4 Department of Neurology, Erasmus MC, University Medical Center Rotterdam, Rotterdam, the Netherlands

**Keywords:** Migraine, Immune system, Inflammation, Headache, Granulocyte, Platelet, Lymphocyte

## Abstract

**Background:**

Although some evidence implicates the immune system in migraine attacks, its role during attack-free periods remains largely unexplored. Therefore, we assessed the association between the immune system and migraine status.

**Methods:**

From the population-based Rotterdam Study, we included 6593 participants who underwent blood sampling and migraine assessments. In the blood samples, we measured white blood-cell-based immune markers. As a marker for the innate immune system, granulocyte and platelet counts were determined, whereas lymphocyte counts were used as a marker for the adaptive immune system. Migraine was assessed using a validated questionnaire based on ICHD-2 criteria. We investigated associations between blood-cell counts and migraine using logistic regression models adjusting for age, sex and other variables.

**Results:**

Mean age of participants was 65.6 ± 11.2 years and 56.7% were female. The lifetime prevalence of migraine was 15.1% (995/6593). We found no statistically significant associations between granulocyte (odds ratio [OR] per standard deviation increase 1.01 95% Confidence Interval [CI]: 0.93–1.09), platelet (OR 1.01 CI: 0.94–1.09) or lymphocyte counts (OR 1.01 CI: 0.93–1.08) and migraine status.

**Conclusions:**

Our results do not support an association between white blood-cell-based immunity markers and migraine status.

## Background

Increasing evidence suggests a role of the immune system in the multi-factorial etiology of migraine attack [1–3]. Compared to healthy individuals, in patients admitted to the hospital during a migraine attack, higher levels of neutrophil and platelet, and lower levels of lymphocyte counts were reported [4–8]. However, other studies failed to replicate these findings [9]. Moreover, some evidence supports long-lasting alterations in some of the pro-inflammatory cytokines (increased TNF-α, IL-1β, IL-6 and IL-8) and lymphocyte subsets (increase in CD4+, decrease in CD8+) in the peripheral blood of migraine patients [10].

The immune system can be broadly categorized into innate and adaptive immunity. In brief, innate immunity could be considered the “first defence” while adaptive immunity refers to an acquired response against specific pathogens [11]. Recent work from the field of oncology shows that easily obtainable white blood-cell-type markers can reliably capture the activity levels of innate and adaptive immunity [12]. Granulocyte (including the most abundant subtype neutrophil) and platelet counts are used as a proxy for the activity of innate immunity, whereas lymphocyte count is used as a proxy for the activity of adaptive immunity [13, 14].

However, previous findings on these markers and migraine mainly come from hospital-based studies, which may exclusively include patients with severe enough complaints warranting a neurological consultation. Furthermore, results from large-scale survey studies indicate that less than half of individuals suffering from migraine ever visit a healthcare professional [15, 16]. Hence, hospital-based studies may suffer from selection bias. Furthermore, we need to distinguish acute responses of the immune system to migraine attacks from the longer-lasting chronic inflammatory responses. However, epidemiological data on the white blood-cell markers during attack-free periods in migraine is lacking. Therefore, in this study, we investigated the association between white blood-cell-based immunity markers and migraine in a population-based cohort.

## Methods

### Study setting and population

The present study was based on the Rotterdam Study which is a prospective population-based cohort study of middle-aged to elderly community residents of the Ommoord district in Rotterdam, The Netherlands. The original cohort of the study (first) started in 1990 followed by an expansion cohort (second) in 2000, both cohorts included participants ≥ 55 years old. In 2006, Rotterdam Study expanded with another cohort (third) which included participants ≥ 45 years old. Once recruited into the study, participants were examined at baseline and subsequently invited for follow-up rounds, including a home interview and visits to a dedicated research centre located in Ommoord every three to six years [17].

The Rotterdam Study has been approved by the Medical Ethics Committee of the Erasmus MC (registration number MEC 02.1015) and by the Dutch Ministry of Health, Welfare and Sport (Population Screening Act WBO, license number 1071272-159521-PG). The Rotterdam Study Personal Registration Data collection is filed with the Erasmus MC Data Protection Officer under registration number EMC1712001. The Rotterdam Study has been entered into the Netherlands National Trial Register (NTR; www.trialregister.nl) and into the WHO International Clinical Trials Registry Platform (ICTRP; https://apps.who.int/trialsearch/) under shared catalogue number NTR6831. All research was performed in accordance with relevant guidelines/regulations. All participants provided written informed consent to participate in the study and to have their information obtained from treating physicians.

### Laboratory assessments

Fasting blood samples were collected during a visit to the research center approximately one week after the migraine interview. Complete blood count measurements were conducted with the COULTER® Ac-T diff2™ Hematology Analyzer (Beckman Coulter, Brea, CA, US) directly after the blood sample was drawn. Laboratory measurements included absolute granulocyte, platelet, and lymphocyte counts in 10^9^ per liter [12].

### Assessment of migraine

Migraine was assessed during a home interview or over the phone (due to logistic reasons, n = 634) using a validated questionnaire by trained interviewers. The questionnaire was based on the modified International Classification of Headache Disorders, second edition (ICHD-II) criteria [18], and validated for use in the Genetic Epidemiology of Migraine study [19], with a sensitivity of 0.93 and a specificity of 0.36. The lifetime occurrence of migraine attacks was assessed using the following diagnostic criteria: having at least five attacks of severe pain lasting 4–72 h (when untreated) with a minimum of two of the four characteristics (unilateral location; pulsating quality; severe pain intensity; worsening by or causing avoidance of routine physical activity), and any of the two characteristics (nausea; photophobia and phonophobia), with these symptoms not being attributed to another disorder. Participants who met the criteria for lifetime history of migraine were categorized as persons with migraine. Moreover, migraine with aura was defined as meeting all the criteria for migraine without aura and additionally reporting headache attacks with visual, sensory, or language-related aura symptoms which lasted between 5 and 60 min. Also, to differentiate history of migraine from current migraine at the time of baseline interview, we categorized individuals as having active migraine (the last attack occurred less than one year ago) or non-active migraine (the last attack occurred more than one year ago).

As described before [20], the criteria used in our questionnaire were slightly different from the ICHD-2 criteria in two ways: First, only participants who had reported a history of severely (instead of moderately or severely) painful headaches received the complete set of questions (i.e., the first question served as a screening tool). Therefore, persons suffering from migraine with moderately painful headaches and persons who had aura without migraine headache would be categorized as participants without migraine. Second, migraine with aura was defined as reporting five (instead of two) headache attacks.

### Covariables

Information on other variables was collected via interview, blood sampling and physical examination: body mass index (weight in kg divided by height in m^2^), hypertension (blood pressure systolic ≥ 140 mm Hg, diastolic ≥ 90 mm Hg or blood pressure lowering medication use) [21], high-density lipoprotein (HDL) cholesterol [21], C-reactive protein levels (measured via particle enhanced immunoturbidimetric assay, Roche Diagnostics, Mannheim, Germany), diabetes mellitus (fasting glycaemia ≥ 7.0 mmol/L or glucose-lowering medication use) [22], education level (primary; lower/intermediate education; intermediate vocational or higher general education; and higher vocational education or university) and smoking status (never, former and current smoker).

### Statistical analyses

We quantified the association of the different blood-cell counts with the lifetime prevalence of migraine using the following strategy. First, all types of blood-cells were standardized to establish comparability across different scales. Second, we constructed logistic regression models to estimate the association between immunity markers and the prevalence of migraine. In model 1 we adjusted for age and sex. In model 2, we additionally adjusted for education level, smoking status, body mass index, diabetes mellitus, HDL levels, hypertension and study cohort. Third, we analysed the associations between blood-cell counts and migraine subgroups (migraine with aura and active migraine) using multinomial logistic regression (reference group was the participants without migraine) with the adjustment for the same covariables as in model 2. We also fitted separate models where we adjusted blood-cell-type counts for each other (e.g., granulocytes corrected for platelets and lymphocytes). Finally, we conducted a sensitivity analysis to see if the results were affected by a current infection (increase in CRP levels). Analyses were repeated after excluding individuals with high CRP levels (> 10 mg/L).

IBM SPSS version 25 [23] and R version 4.0.5 [24] for Windows were used for the analyses. Missing values among covariables were imputed using multiple imputations by chained equations based on all variables but migraine and immune markers, with 5 imputations and 10 iterations using the “mice” package (version 3.13.0) in R [25]. The fitness of the models was evaluated through Akaike Information Criterion metric.

## Results

A total of 7266 participants were interviewed to ascertain their lifetime history of migraine. For 673 of the participants, blood tests were not collected due to logistic reasons and/or reluctance for blood collection. This left us with a sample of 6593 participants with both migraine status and data on relevant immunity markers available. Table 1 shows the descriptive characteristics of the study population. In total, 995 (15.1%) participants had a history of lifetime migraine. Among those, 202 (20.3%) had migraine with aura and 391 (39.3%) had active migraine. 80.9% of migraine patients were female and the participants with migraine were younger compared to participants without migraine (median age 62.0 vs. 66.7).

**Table 1:** Descriptive Characteristics of the study population

	Overall (N = 6593)	Lifetime history of migraine	
		No (N = 5598)	Yes (N = 995)
**Cohort**			
First	1460 (22.1)	1282 (22.9)	178 (17.9)
Second	1594 (24.2)	1358 (24.3)	236 (23.7)
Third	3539 (53.7)	2958 (52.8)	581 (58.4)
**Female**	3739 (56.7)	2934 (52.4)	805 (80.9)
**Age, years**	66.11 [18.33]	66.70 [18.48]	61.95 [17.33]
**Educational Level**			
Primary	613 (9.3)	522 (9.3)	91 (9.1)
Intermediate	2595 (39.4)	2154 (38.5)	441 (44.3)
Higher	1944 (29.5)	1694 (30.3)	250 (25.1)
University	1441 (21.9)	1228 (21.9)	213 (21.4)
**Migraine with Aura**	202 (3.1)	*NA*	202 (20.3)
**Active Migraine**	391 (5.9)	*NA*	391 (39.3)
**CRP, mg/L**	1.30 [2.30]	1.30 [2.30]	1.20 [2.50]
**Blood-cell-type counts, 10** ^**9**^ **/L**			
Granulocyte	3.90 [1.70]	4.00 [1.70]	3.80 [1.50]
Lymphocyte	2.30 [0.90]	2.30 [0.90]	2.30 [0.90]
Platelet	270.00 [85]	268.00 [85]	280.00 [85]
**BMI, kg/m2**	27.62 ± 4.39	27.61 ± 4.33	27.72 ± 4.72
**Diabetes Mellitus**	728 (11.0)	647 (11.6)	81 (8.1)
**HDL cholesterol, mmol/l**	1.45 ± 0.42	1.44 ± 0.42	1.51 ± 0.42
**Hypertension**	4427 (67.1)	3781 (67.5)	646 (64.9)
**Smoking**			
Former	3290 (49.9)	2797 (50.0)	493 (49.5)
Current	1111 (16.9)	972 (17.4)	139 (14.0)

Values are presented either as mean ± Standard Deviation, as median [interquartile range] or as number (%). Number of missing values before imputation: 3 (0.05%) for smoking, 60 (0.91%) for educational level, 64 (0.97%) for hypertension, 27 (0.41%) for HDL cholesterol, 19 (0.29%) for body mass index, and 12 (0.18%) for diabetes mellitus. BMI, body mass index; HDL, high-density lipoprotein; CRP, C-Reactive Protein

We found no association of granulocyte counts (adjusted odds ratio [OR] per standard deviation increase 1.01 95% Confidence Interval [CI]: 0.93–1.09), platelet (OR 1.01 CI: 0.94–1.09) and lymphocyte counts (OR 1.01 CI: 0.93–1.08) with migraine status. Results did not change when blood-cell counts were corrected for each other (see Table 2). In addition, adjusted multinomial logistic regression models showed no significant associations between blood-cell counts and active/non-active migraine status (Table 3). This was also true when blood-cell counts were corrected for each other. One exception was that platelet count and having a migraine with aura were positively associated (per one standard deviation increase in platelet count, adjusted odds ratio [OR] 1.16, 95% Confidence Interval: [CI] 1.01–1.33). This was also true when platelet counts were corrected for granulocyte and lymphocyte counts (Adjusted OR 1.18, CI: 1.02–1.36; see Table 3). Finally, after excluding participants with high CRP levels (> 10 mg/L) the results did not change.

**Table 2:** Logistic regression analyses for the association between blood-cell counts and migraine

Laboratory assessment	Any Migraine (n/N = 995/6593)	
	Model 1^a^ OR (95% CI)	Model 2^b^ OR (95% CI)
Granulocytes	0.98 (0.91– 1.05)	1.01 (0.93–1.09)
Corrected for platelets and lymphocytes	0.97 (0.90–1.05)	1.01 (0.93–1.09)
Platelets	1.01 (0.94–1.08)	1.01 (0.94–1.09)
Corrected for granulocytes and lymphocytes	1.02 (0.94–1.09)	1.01 (0.94–1.09)
Lymphocytes	0.99 (0.91–1.06)	1.01 (0.93–1.08)
Corrected for granulocytes and platelets	0.99 (0.91–1.07)	1.01 (0.92–1.08)

Model 1 is adjusted for age and sex. Model 2 is adjusted for age, sex, education, smoking status, body mass index, diabetes mellitus, HDL cholesterol, hypertension and study cohort. All types of blood were standardized. OR, odds ratio; CI, confidence interval. (a) AIC: 5283. (b) AIC: 5275.

**Table 3:** Multinomial logistic regression analyses for the association between blood-cell counts and migraine subgroups

Laboratory assessment	Odds Ratio (95% CI)	
	Non-active Migraine^a^ (n/N = 604/995)	Active Migraine^a^ (n/N = 391/995)
Granulocytes	0.99 (0.90– 1.09)	1.02 (0.91– 1.15)
Corrected for platelets and lymphocytes	0.97 (0.88–1.08)	1.04 (0.92–1.17)
Platelets	1.03 (0.94–1.12)	1.00 (0.89–1.12)
Corrected for granulocytes and lymphocytes	1.03 (0.94–1.13)	1.00 (0.89–1.13)
Lymphocytes	1.05 (0.97–1.13)	0.90 (0.78–1.05)
Corrected for granulocytes and platelets	1.05 (0.97–1.13)	0.90 (0.77–1.04)
	**Migraine without Aura** ^**b**^ **(n/N = 793/995)**	**Migraine with Aura** ^**b**^ **(n/N = 202/995)**
Granulocytes	1.02 (0.94–1.11)	0.96 (0.82–1.13)
Corrected for platelets and lymphocytes	1.03 (0.94–1.12)	0.91 (0.77–1.09)
Platelets	0.98 (0.90–1.06)	1.16 (1.01–1.33)
Corrected for granulocytes and lymphocytes	0.97 (0.89–1.06)	1.18 (1.02–1.36)
Lymphocytes	1.01 (0.93–1.10)	1.01 (0.85–1.19)
Corrected for granulocytes and platelets	1.01 (0.92–1.10)	0.99 (0.83–1.19)

Models are adjusted for age, sex, education, smoking status, body mass index, hypertension, HDL cholesterol, diabetes mellitus and study cohort. Reference: participants without migraine. All types of blood-cells were standardized. CI, confidence interval. (a) AIC: 6413. (b) AIC: 6297.

## Discussion

In this study, we did not find an association between markers of the immune system such as granulocyte, platelet and lymphocyte counts, and migraine status.

Previous findings on blood-cell-type-based markers during migraine attack are inconclusive. In summary, some of the studies reported a higher neutrophil [4–6], platelet [5] counts, and lower lymphocyte counts [4, 5] in migraine patients during the ictal period compared to healthy controls. However, these findings were not replicated by other studies [5–7, 9]. In addition, previous studies were hospital-based and conducted within a selected group of participants suffering from migraine attacks severe enough to warrant admission to a hospital. This may potentially introduce selection bias in some of the hospital-based studies. In the present study, we measured the immune markers in general population, during headache-free period. Within the complex pathophysiology of migraine, among others, activation of trigeminovascular neurons results in the release of substances such as calcitonin gene-related peptide (CGRP), substance P, nitrous oxide, vasoactive intestinal peptide and serotonin [26, 27]. These changes may in turn initiate a cascade of processes including mast cell degranulation, vasodilation, microglia activation and leukocyte infiltration leading to inflammatory reactions in the absence of a pathogen (i.e. neurogenic inflammation) [28]. Since this neurogenic inflammation occurs during the migraine attack, the immune reaction by leukocytes and consecutive changes in these blood-cell-type counts (increase in granulocyte and platelet, decrease in lymphocyte) are expected to occur during the ictal period. Although the acute response of the immune system during migraine attacks has been studied, long-lasting chronic inflammatory response is largely unknown. One study on 22 migraine patients found alterations in lymphocyte subsets (increase in CD4+, decrease in CD8+) in the peripheral blood of migraine patients outside the attacks [29], but this finding was not replicated in other studies. Our current findings do not support long-lasting chronic immune-inflammatory differences between participants with and without migraine.

Of note, in the sub-analyses, we observed that platelet count was weakly associated with having a migraine with aura. Sensitized platelet metabolism may play a role in migraine with aura pathophysiology [30]. As this observation was made in a sub-analysis, we would like to highlight that further confirmation is needed from other studies, before we can draw firm conclusions regarding this association.

### Strengths and limitations

There are several strengths of this study. Firstly, we used a validated migraine questionnaire which reduces the risk of misdiagnosis. Secondly, by including participants from the general population, we could additionally capture the migraine patients who did not visit a hospital, reducing the risk of selection bias. Thirdly, we measured and corrected our findings for a variety of potential confounders. Meanwhile, we also acknowledge some limitations of this study. First, there is a possibility that migraine patients with less than severe headache may have been misclassified as participants without migraine since we used a modified version of the ICHD-2 criteria for migraine assessment. It can be argued that this may reduce the contrast between the groups, explaining the absence of difference in terms of immune system markers. However, we believe this modification would not results in a differential misclassification bias since it is unrelated to immune system markers. Second, the cross-sectional nature of our analysis makes it difficult to understand the temporal relationship between blood markers and migraine. Third, the questionnaire was designed at a time when ICHD-2 criteria were used widely, however, the current diagnostic standard is ICHD-3 beta. In these revised criteria, in addition to visual, sensory and speech/language-related symptoms, there are also motor-, brainstem-related and retinal aura symptoms. Although we had insufficient information about these symptoms, these are considerably less frequently seen than the symptoms which we included [31] therefore we believe the risk of (non-differential) information bias is low. Fourth, it would be interesting to test the effect of the temporality of the last attack relative to the blood sampling. However we do not have data on the exact dates of attacks or attack frequency. Similarly, this lack of information prevents us from comparing blood markers during and in-between attacks. Fifth, post-hoc analysis showed that participants without blood samples (*n* = 673), had overall worse health profile but lower prevalence of migraine. Worse health profile could potentially be related to exposure (immunity markers) and thus we could be facing missingness not at random. However, as the prevalence of migraine is lower in participants without blood samples than in those with blood samples, we do not think that the addition of these samples would have led to significant associations between immunity markers and migraine. Finally, we investigated the general immune response with a select group of blood-cell-type counts, however, we cannot completely exclude the possibility that specific immune markers and inflammatory processes are involved in the migraine pathophysiology.

In conclusion, we did not find evidence for an association between white blood-cell-based immunity markers and migraine status.

## Data Availability

The datasets analysed during the current study are not publicly available due restrictions based on privacy regulations and the informed consent of the participants, but are available from the data management team of the Rotterdam Study (datamanagement.ergo@erasmusmc.nl) on reasonable request, which has a protocol for approving data requests.

## Abbreviations

BMI: Body mass index
CD4+: CD4-positive cells
CD8+: CD8-positive cells
CGRP: Calcitonin gene-related peptide
CRP: C-reactive protein
HDL: High-density lipoprotein
ICHD: International Classification of Headache Disorders
IL-1β: Interleukin 1 beta
IL-6: Interleukin 6
IL-8: Interleukin 8
TNF-α: Tumor necrosis factor alpha
